# SARS-CoV-2 immune responses in patients with multiple myeloma and lenalidomide maintenance therapy

**DOI:** 10.3389/fimmu.2024.1510942

**Published:** 2024-12-18

**Authors:** Ioana Martac, Sina A. Beer, Aileen Schenk, Osama Ahmad, Claus-Philipp Maier, Gülay Demirel, Beate Preuß, Reinhild Klein, Anna M. P. Stanger, Britta Besemer, Luca Hensen, Claudia Lengerke

**Affiliations:** ^1^ Department of Internal Medicine II, Hematology, Oncology, Clinical Immunology and Rheumatology, University Hospital Tübingen, Tübingen, Germany; ^2^ Department of General Pediatrics, Hematology/Oncology, Children’s University Hospital Tuebingen, Tübingen, Germany

**Keywords:** multiple myeloma, immunomodulatory therapy, lenalidomide, cellular immune responses, vaccine response

## Abstract

**Introduction:**

Multiple myeloma (MM) is an uncontrolled plasma cell proliferation in the bone marrow, leading to immune dysregulation with impaired humoral immune responses. Conversely, cellular-based responses play a vital role in MM patients. However, the extent and duration of cellular-induced protection remain unclear to date. Here, immunomodulatory drugs (IMiDs) like Lenalidomide (Lena) become interesting, as they may have stimulatory effects on T-cell functioning.

**Methods:**

In this study we investigated immune responses elicited by COVID-19 vaccine or infection comparing 43 healthy volunteers (avg. 35y, 72.1% female, 81.4% previously COVID-19 infected), with 41 MM patients under Lena maintenance therapy (avg. 63.8y, 51.2% female, 61% previously COVID-19 infected). Humoral responses to SARS-CoV-2 spike (S), spike-RBD, and nucleocapsid (N) were measured via ELISA in subjects’ plasma. Freshly isolated PBMCs, incubated with SARS-CoV-2 peptides (N, S), activation induced marker (AIM) assays and flow cytometry, allowed us to assess cellular responses (CD8^+^ T, T_(F)H_: CD4^+^ T (follicular) helper).

**Results:**

Whereas healthy controls showed significant better humoral responses (N IgA p<0.001), T cell responses were robust in the MM group (higher S-act. T_H_, p<0.001). Stratified by COVID-19 status, the MM group showed higher N-act. T_H_ (p=0.03). These results were unchanged comparing a Lena intake with Lena break around vaccination.

**Discussion:**

Taken together, MM patients under Lena therapy exhibit weakened antibody production but present a robust T cell response following SARS-COV-2 infection or vaccination. Our results highlight the importance of vaccination in this subgroup and moreover, argue against a Lena intake break around the time of vaccination.

## Highlights

- Despite lower antibody levels, T cell responses to SARS-CoV-2 vaccination in multiple myeloma patients are comparable to healthy controls.
- Taking a break from immunomodulatory therapy did not enhance vaccine responses in patients.

## Introduction

Multiple myeloma (MM) is characterized by an uncontrolled proliferation of clonal plasma cells in the bone marrow (BM), leading to immune dysregulation (1). Prolonged drug administration in these patients may further impair immunity and contribute to an enhanced risk for infections (2). The COVID-19 pandemic has highlighted this problem, as high infection and mortality rates were particularly reported in this population (3–7). While MM therapy is more effective than ever, we face a significant B cell depletion using most treatments. Weaker humoral immune responses (2, 8, 9), and drastically reduced seroconversion rates following exposure to a viral antigen via infection or vaccination were especially observed in patients receiving combined treatment regimens (10, 11). In healthy individuals, humoral and cellular responses to infection or vaccination are equally represented (12). Instead, patients with MM may rely on protection through cellular-based responses (13–15) to compensate insufficient humoral reactions (16, 17), although the current data remain inconclusive (18). Since next to antibodies, mRNA-based vaccines also induce CD4^+^ T cells as well as CD8^+^ T cells responses (19), they may offer effective protection in individuals with impaired humoral immunity such as patients with MM. However, cellular immune responses could be further negatively influenced by other patient- or treatment related factors such immune dysfunction or lymphopenia associated with the presence of cancer cells in the body, anti-cancer treatments, or advanced age (13, 20).

Interestingly, treatment effects may not be in all cases negative. Immune checkpoint-inhibiting molecules, or Lenalidomide (Lena), an immunomodulatory drug (IMiD) frequently used in MM, may in fact rather stimulate immune cells (21), and thus also augment immune responses to pathogens. The unpredictability of a cellular response in the context of a low or absent humoral response are however a great concern of patients as well as treating physicians. In this study, we investigate humoral and cellular responses elicited by COVID-19 vaccine or SARS-COV-2 infection in MM patients with Lena maintenance therapy in comparison to healthy individuals, to explore the effects on immune responses.

## Patients and methods

### Study population and clinical data acquisition

The study included MM patients treated with ongoing or prior Lena maintenance therapy between 05/2022 and 10/2023 in the Department of Internal Medicine II of the University Hospital Tübingen. Healthy volunteers served as controls. In both groups, HIV- or hepatitis C infections as well as a hemoglobin level < 10 g/dl were exclusion criteria due to ethics regulations and the aim to reduce confounding factors. Healthy controls were additionally excluded if any history of immunosuppression, e.g. long-term glucocorticoids, was reported. After study inclusion, all study participants completed a questionnaire (**Supplementary Material 1**) that addressed pre-existing conditions, medication intake (for MM patients: duration and intake break of Lena), COVID-19 vaccinations, and COVID-19 infections (tested by antigen rapid test or PCR) with its subjective severity. A Lena intake break was defined as 7 days break in sum before and/or after the vaccination. For MM patients, electronic medical records were reviewed regarding disease subtype, therapy lines, remission status [defined based on the international myeloma working group (IMWG) criteria (22)], neutropenia (defined <1000/µl), lymphopenia (defined <500/µl), quantitative immunoglobulin levels (severe hypogammaglobulinemia defined <400 mg/dl), Lena dosage per day, as well as supportive intravenous immunoglobulin (IVIG) therapy, using the following hospital databases. The study was approved by the institutional Ethical Committees in accordance with the Declaration of Helsinki (no. 143/2022BO2). All patients and controls provided informed consent before study inclusion.

### Analysis of SARS-CoV-2 T cell responses

SARS-CoV-2-specific CD8^+^ and CD4^+^ T cell responses were examined. T cell activation (act.) was measured using an activation induced marker (AIM) assay (CD69^+^CD137^+^ for CD8^+^ and CD25^+^CD134^+^ for CD4^+^ T cells) on fresh isolated peripheral blood mononuclear cells (PBMCs). PBMCs were isolated in Ficoll-Paque *(density 1.077 g/ml, PAN biotech)* using Sepmate tubes. The cells were resuspended in culture medium, consisting of 5% autologous plasma and 95% RPMI *(RPMI-1640 +Penicillin G/+Streptomycin sulfate, Gibco)* and incubated in 96 well plates up to 24 hours at 36°C and 5% CO_2_ concentration with different SARS-CoV-2 peptides (N*: PepTivator SARS-CoV-2 Prot_N 1*μg/ml*, 130-126-698, Miltenyi Biotec, GenBank MN908947.3, Protein QHD43423.2;* S: *PepTivator SARS-CoV-2 Prot_S Complete 1*μg/ml*, 130-127-951, Miltenyi Biotec, GenBank MN908947.3, Protein QHD43416.1)*, resulting in T cell responses directed against the total S protein and N. As positive control we performed phytohemagglutinin (PHA, 5μg/ml) stimulation. Sterile water served as negative control. Its values were subtracted from all measurements to eliminate background interference. Surface staining with anti-human antibodies *(Miltenyi Biotec*, **Supplementary Table S1**) enabled the detection of activated S CD8^+^ T cells, CD4^+^ T follicular helper (T_FH_) and CD4^+^ T helper cells (T_H_ cells) via flow cytometry (*BD FACSLyric)*. The specimens were fixed using a 2% solution of paraformaldehyde (PFA) prior to assessment. A positive T cell response was determined by a magnitude equal to or greater than 0.1%.

### Analysis of SARS-CoV-2 antibody responses

To explore immune responses elicited by COVID-19 vaccine or SARS-CoV-2 infection, we employed a two-pronged, non-quantitative approach. First, we determined antibody responses (IgA and IgG) by ELISA from subjects´ plasma against Spike (S) 1 protein *(recombinant SARS-CoV-2 Spike Protein, target concentration 0.1 µg/ml, Sino Biological)*, spike-receptor binding domain (RBD) *(SARS-CoV-2 Spike protein (RBD)-His tag, target concentration 0.3 µg/ml, GenScript)* and Nucleocapsid (N) *(recombinant SARS-CoV-2 (2019-nCoV) Nucleocapsid Protein-His tag, target concentration 0.1 µg/ml, Sino Biological)*. COVID-19 infection status was detected by anti-N IgG antibodies and verified by spike-RBD ELISA. Test antigens were diluted with bicarbonate buffer. Nunc plates were coated with a dilution of test antigen, blocked with a wash buffer containing PBS (phosphate-buffered saline) and BSA (bovine serum albumin), and subsequently incubated with anti-human antibodies (IgG and IgA, respectively), followed by the addition of substrate (citrate buffer, O-Phenylenediamine, and Hydrogen Peroxide). Control sera were used as comparison. Antibody responses were quantified in arbitrary units relative to the control sera. The cut-off thresholds, indicative for both positive reactivity and onset of seroconversion, were calculated as the sum of the mean value plus three times the standard deviation of control sera (**Table 1**). Additionally, we defined a seroconversion rate based on Spike-1 IgG responses.

**Table 1:** Determined cut-off thresholds for positive antibody results in the performed ELISAs.

Antibody target and isotype	Cut-off [arbitrary units]
Spike-1 IgG (= seroconversion)	> 15.0
Spike-1 IgA	> 10.0
Spike-RBD IgG	> 6.0
Spike-RBD IgA	> 15.0
Nucleocapsid IgG	> 15.0
Nucleocapsid IgA	> 15.0

### Statistical analysis

Statistical analyses were performed using the Software Excel (Microsoft Office Professional Plus v. 2312) and GraphPad PRISM version 9.4.1 (681). Patient characteristics were expressed as frequencies or categorical variables. Categorical data was compared via Chi-squared-test (χ2) or exact Fisher-test. Continuous variables were further statistically examined using the Mann-Whitney-U-test to compare experimental data (antibody levels, T cell responses) in relation to clinical data (age, gender, diagnosis, and treatment status). Correlations between antibody and T cell responses were computed using Spearman correlation. Due to the limited sample size, non-parametric tests were used in most instances. All tests of significance were two-sided, and a p-value<0.05 was considered significant. When using boxplots for visualization, we displayed mean values as horizontal lines within the bars. Outliers are represented as individual points. Significant differences between groups are graphically indicated by asterisks: single asterisk (*) = significance level of p<0.05, double asterisk (**) = significance level of p<0.01.

## Results

### Study population, patient characteristics and treatment history

Forty-three healthy controls and forty-one MM patients (MM subtype: n=24 IgG kappa, n=7 light chain MM, n=5 IgA kappa, n=5 IgG/IgA lambda) with prior or ongoing Lena maintenance therapy were included. Healthy controls were on average 35 years old (range 21-61) and 72.1% (n=31) were female. The average age of MM patients was 63.8 years (range 47-78) and 51.2% (n=21) were female. MM patients were significant older compared to healthy controls (p<0.001).

At enrollment, 73% (n=30) MM patients were in complete serological remission (CR), 7.3% (n=3) showed a very good partial response (VGPR), loss of CR, partial remission (PR) with stable disease (SD), and 4.8% (n=2) had progressive disease (PD). In line, thirty-nine out of 41 patients were still on 1^st^ line treatment. Eleven patients received monthly IVIG support. Lab tests at study enrollment showed stable blood counts with neutropenia or lymphopenia in 12.1% (n=5) each, and severe hypogammaglobulinemia in 9.7% (n=4) MM patients. All MM patients received proteasome inhibitors (PI) and IMiD-based treatment. The induction therapy combined a triplet (n=13) or, more recently, a quadruplet regimen (n=23, added anti-CD38 in n=16 and anti-SLAMF7 monoclonal antibody in n=7). High dose melphalan chemotherapy followed by autologous stem cell transplantation (HDC/ASCT) was administered to all but one patient. Tandem transplantations were performed in eleven patients, and six patients were less than 1-year post-HDC/ASCT at study enrollment. While the entire group started on Lena maintenance, 82.9% (n=34) were still taking Lena daily. 17% (n=7) discontinued Lena, mainly due to intolerable side effects and presented without myeloma-specific therapy. One patient developed secondary malignancy (malignant melanoma) and discontinued Lena during the nivolumab treatment. The average Lena treatment duration in the whole group was 4.7 years (range 1-23), with an average dosage of 9.85 mg/day (range 5-15 mg/day) in the group with ongoing Lena treatment. Clinical characteristics are summarized in **Table 2**.

**Table 2:** Multiple myeloma group; patient and therapy characteristics.

Characteristic of patients with MM	Shares
Total patients (male/female)	100%, N = 41 (20/21)
MM subtype	
IgG kappa	58.5%, N = 24
Light chain lambda/kappa	17.0%, N = 7
IgA kappa	12.2%, N = 5
IgG or IgA lambda	12.2%, N = 5
Prior type of therapy	
PI-based	97.5%, N = 40
IMiD-based	97.5%, N = 40
Anti-CD38-based	39.0%, N = 16
anti-SLAMF7-based	17.0%, N = 7
Autologous HCT	97.5%, N = 40
Lenalidomide maintenance	82.9%, N = 34
Recent serological response	
CR	73.2%, N = 30
Loss of CR	7.3%, N = 3
VGPR	7.3%, N = 3
SD/PR	7.3%, N = 3
Progress	4.8%, N = 2
IVIG administration	26.8%, N = 11
Intake break (≥ 7 days) around vaccination	34.1%, N = 14
COVID-19 vaccination count	92.7%, N = 38
COVID-19 infection count	60.9%, N = 25

### SARS-CoV-2 vaccination and infection history

**Figure 1:**
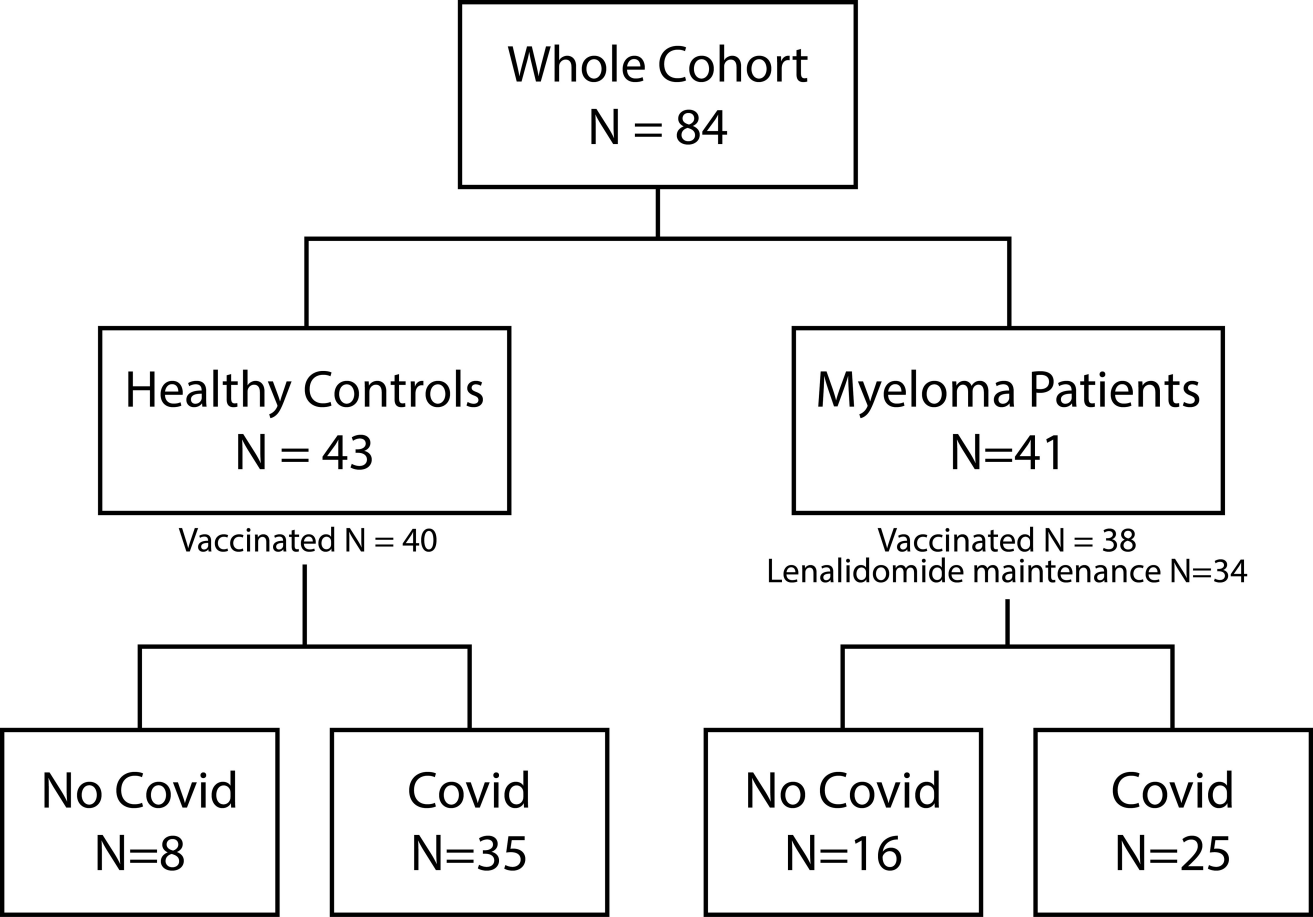
Patient flow. Illustration of the composition of the entire cohort, including healthy controls and Myeloma patients. The number of vaccinated study participants as well as the number of Myeloma patients under Lenalidomide maintenance is depicted. Both study groups can be further divided into no COVID-19 and COVID-19 infection (at least one positive antigen test or PCR).

Forty out of 43 healthy controls (93.0%) and 38 of 41 MM patients (92.6%) received COVID-19 vaccination. 81.4% (n=35) versus 61% (n=25) reported at least one prior COVID-19 infection (p=0.1323). Vaccinations were distributed as follows: In the healthy control group, 81.74% received Comirnaty (1^st^ and 2^nd^ dose BNT162b2 30 µg monovalent or 15/15 µg Original/OmicronBA.4-5 bivalent and Original/Omicron BA.1 15/15 µg bivalent as booster 3^rd^ and 4th), 8.7% Spikevax (1^st^ and second dose mRNA-1273 100 µg and 3^rd^ and 4^th^ dose 50 µg monovalent or 25/25 µg Original/Omicron BA.1 bivalent as booster), 7.83% Vaxzevria (AZD1222 0.5 ml), and 1.74% Jcovden (INN-Ad26.COV2-S 0.5 ml). In the MM group 80.77% received Comirnaty (additional information see above), 15.38% Spikevax, and 3.85% Vaxzevria. Of the 78 vaccinated study participants, 32 of 40 (80%) healthy controls and 24 of 38 (63.16%) MM patients reported at least one SARS-COV-2 infection (**Figure 1**). The time elapsed since the last antigen encounter (vaccination or infection) was considered in statistical analysis, showing no significant difference between the healthy and the MM group (p=0.131 and p=0.209, respectively). MM patients were vaccinated more frequently (median vaccinations 4 vs. 3, p<0.001) compared to healthy individuals. To further homogenize these facts, we additionally performed balanced analyses for vaccination frequency and age. The patient flow is outlined in **Figure 1**.

### Analysis of SARS-CoV-2 T cell responses in the whole cohort

**Figure 2:**
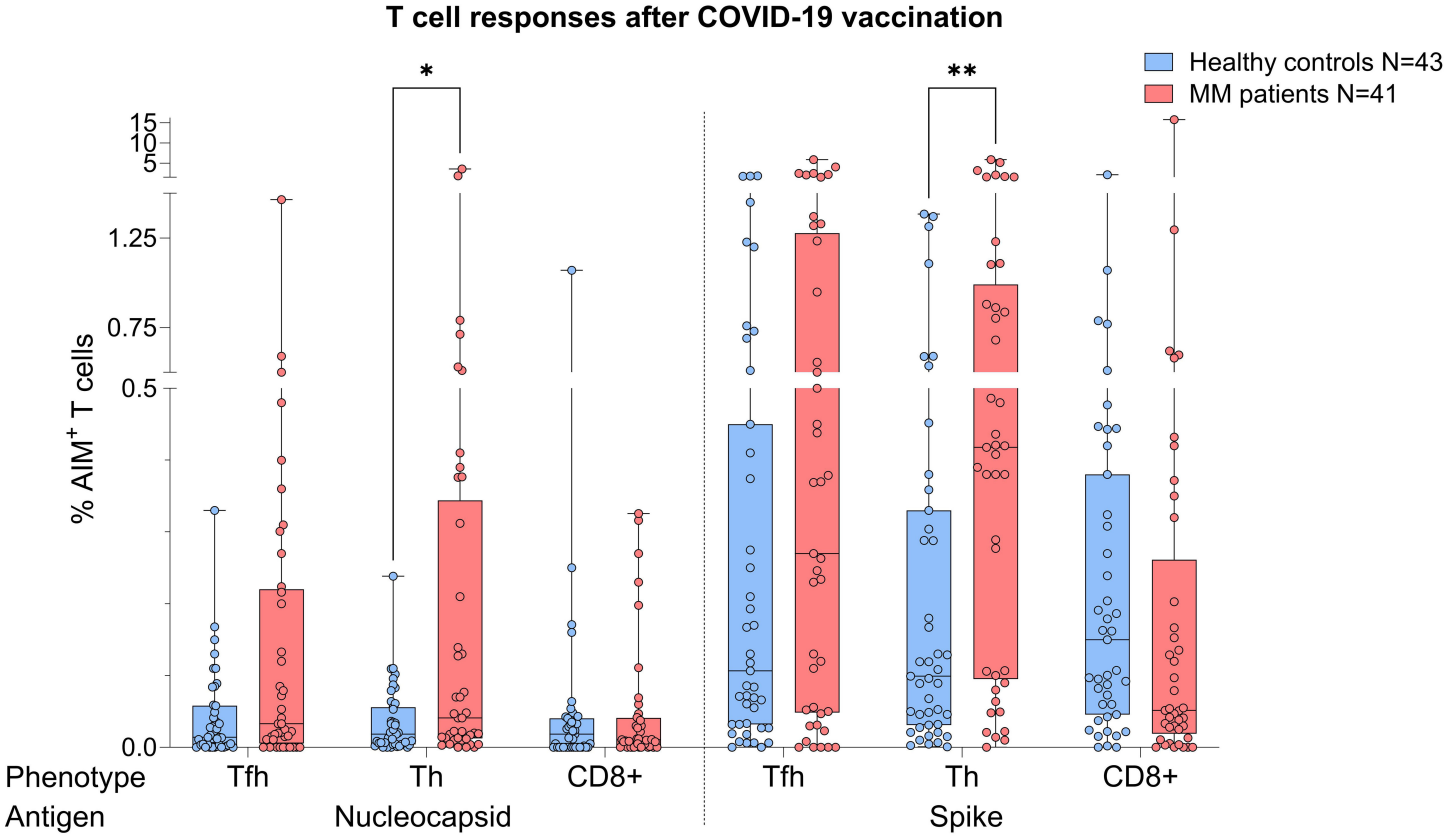
Boxplot comparing T cell responses [CD4^+^ T follicular helper (T_FH_), CD4^+^ T helper cells (T_H_ cells), CD8^+^ T cells] from healthy individuals (control group, in blue) to MM patients with ongoing or prior history of Lena intake (in red), independent of their COVID-19 status. X-axis: T cell activation against SARS-CoV-2 nucleocapsid protein (N) and spike protein (S). Y-axis: respective percentages (%) of activated T cells. In the MM group, significant more S activated (**p=0.0076) and N activated T_H_ cell frequencies (*p=0.043) were seen. Data is represented in a box plot diagram indicating media, Q1 and Q3 with min/max as whiskers.

MM patients showed better T cell responses, independent of COVID-19 status, presenting with higher S act. and N act. T_H_ cell frequencies compared to healthy controls (S: p=0.0076, Mann-Whitney=526.0; N: p=0.043, Mann-Whitney=590.5, **Figure 2**). The PHA positive controls were not showing any significance difference between both T_H_ groups (p=0.368) indicating an antigen-specific difference in response (**Supplementary Figure S1**).

**Figure 3:**
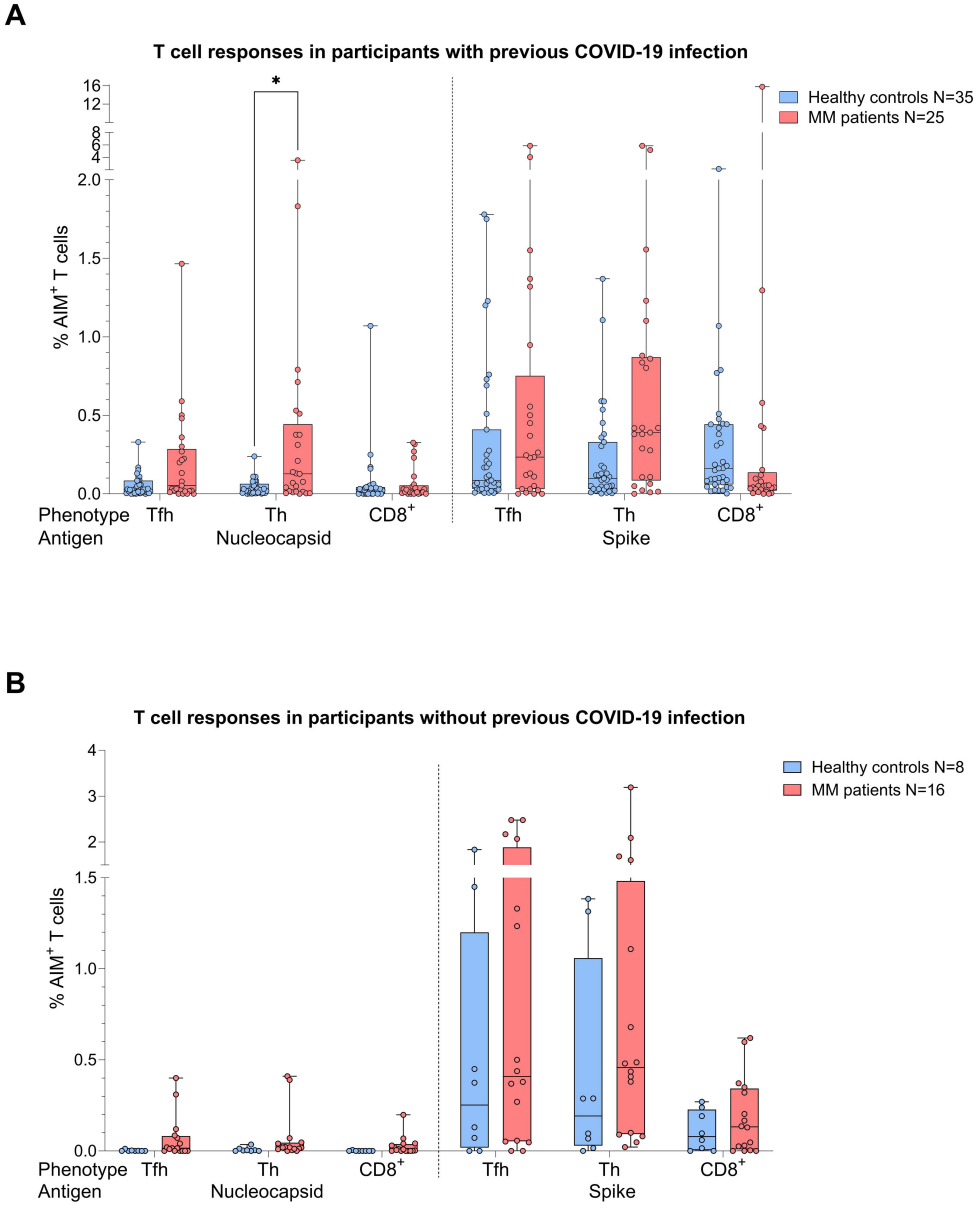
Boxplot comparing T cell responses [CD4^+^ T follicular helper (T_FH_), CD4^+^ T helper cells (T_H_ cells), CD8^+^ T cells] from healthy individuals (control group, in blue) to MM patients with ongoing or prior history of Lena intake (in red), dependent on their COVID-19 status. X-axis: T cell activation against SARS-CoV-2 nucleocapsid protein (N) and spike protein (S). Y-axis: respective percentages (%) of activated T cells. **(A)** (top): COVID-19 group, in the MM group significant more N activated T_H_ cells (*p=0.03) and a trend to significance for S activated T_H_ cells (p=0.056) were seen. **(B)** (bottom): No COVID-19 group, no significant differences in T cell frequencies. Data is represented in a box plot diagram indicating media, Q1 and Q3 with min/max as whiskers.

When the same comparative analysis was performed taking into account the COVID-19 status, act. T cell frequencies were again higher in the MM group (**Figure 3**). Among individuals with prior COVID-19 infection, this difference reached significance for N act. T_H_ cells (p=0.03, Mann-Whitney=252.5, **Figure 3A**), while a trend was observed for S act. T_H_ cells (p=0.056). In the no COVID-19 group, T cell frequencies were by trend superior in the MM patients compared to the control group (**Figure 3B**).

In total, we observed 75.6% and 72.1% T cell responses in the MM and healthy group (frequency of AIM^+^ cells > 0.1%), respectively. In detail, we saw a more dominant S-specific T cell response in the MM patients compared to healthy individuals (73.2% vs 67.4%), whereas N-specific responses were less pronounced (39.0% vs 18.6%). In most cases, the T cell response of our MM patients was triggered by both CD4^+^ and CD8^+^ (48.4%), followed by CD4^+^ dominated responses (45.2%), while few MM patients (6.4%) showed a response solely mediated by CD8^+^. Since MM patients were vaccinated more frequently (median 4 vs. 3), we rechecked T_H_ cell responses by balancing the number of vaccinations in both cohorts. As a result, we had n=27 MM patients and n=30 healthy controls with an equal median vaccination count of 3. Using the same statistical procedures, we were now unable to identify any group differences regarding T cell frequencies against N (T_FH_ p=0.274, T_H_ p=0.179, CD8^+^ p=0.901) or S (T_FH_ p=0.463, T_H_ p=0.072, CD8^+^ p=0.463), *independent* of their COVID-19 status. T cell responses were comparable between healthy and MM individuals.

### Analysis of SARS-CoV-2 antibody responses in the whole cohort

**Figure 4:**
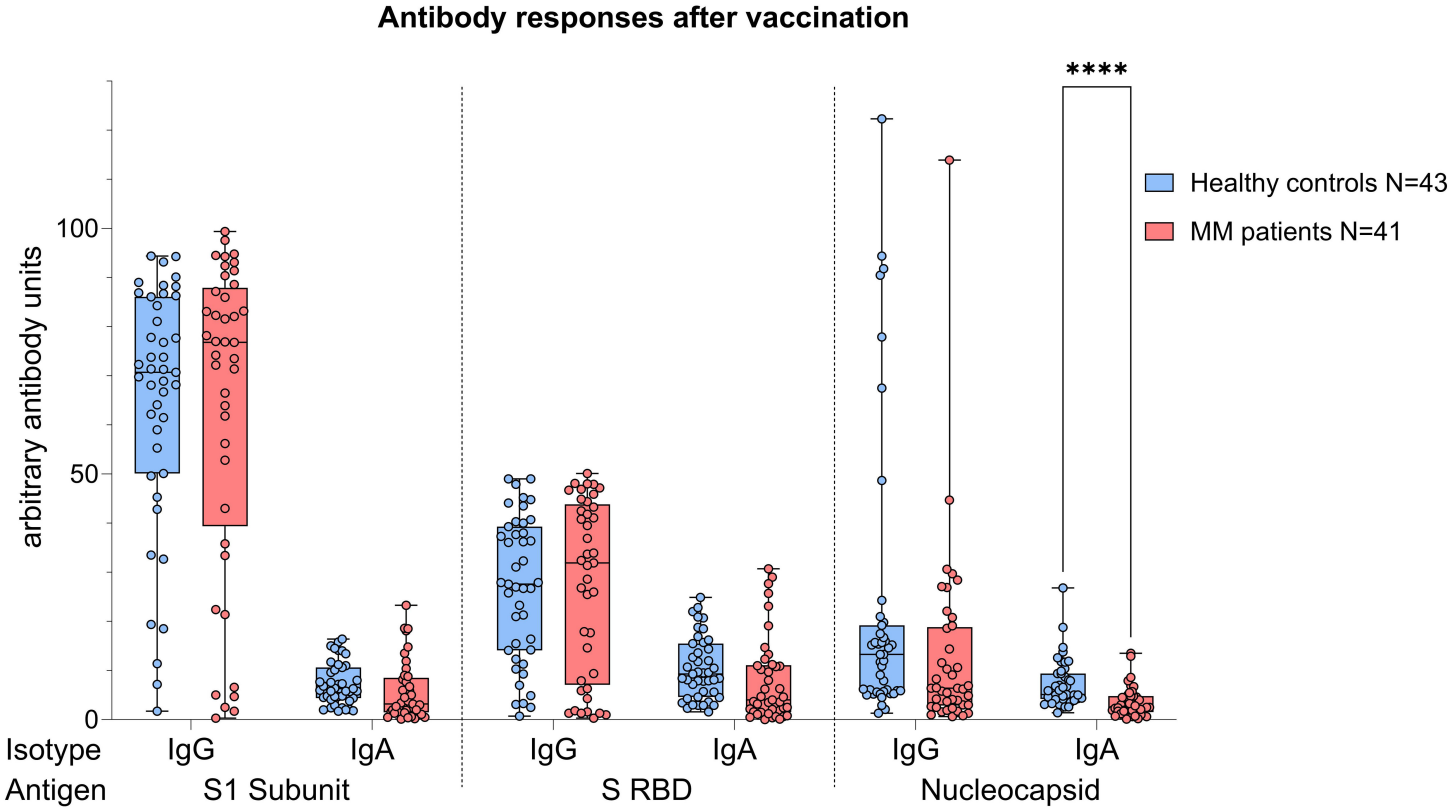
Boxplot comparing antibody responses [IgA, IgG] from healthy individuals (control group, in blue) to MM patients with ongoing or prior history of Lena intake (in red), independent of their COVID-19 status. X-axis: antibody against SARS-CoV-2 spike protein (S1), spike-RBD and nucleocapsid protein (N). Y-axis: arbitary units of antibody levels. The control group presented with significant higher N IgA levels (****p<0.001). Data is represented in a box plot diagram indicating media, Q1 and Q3 with min/max as whiskers.

**Figure 5:**
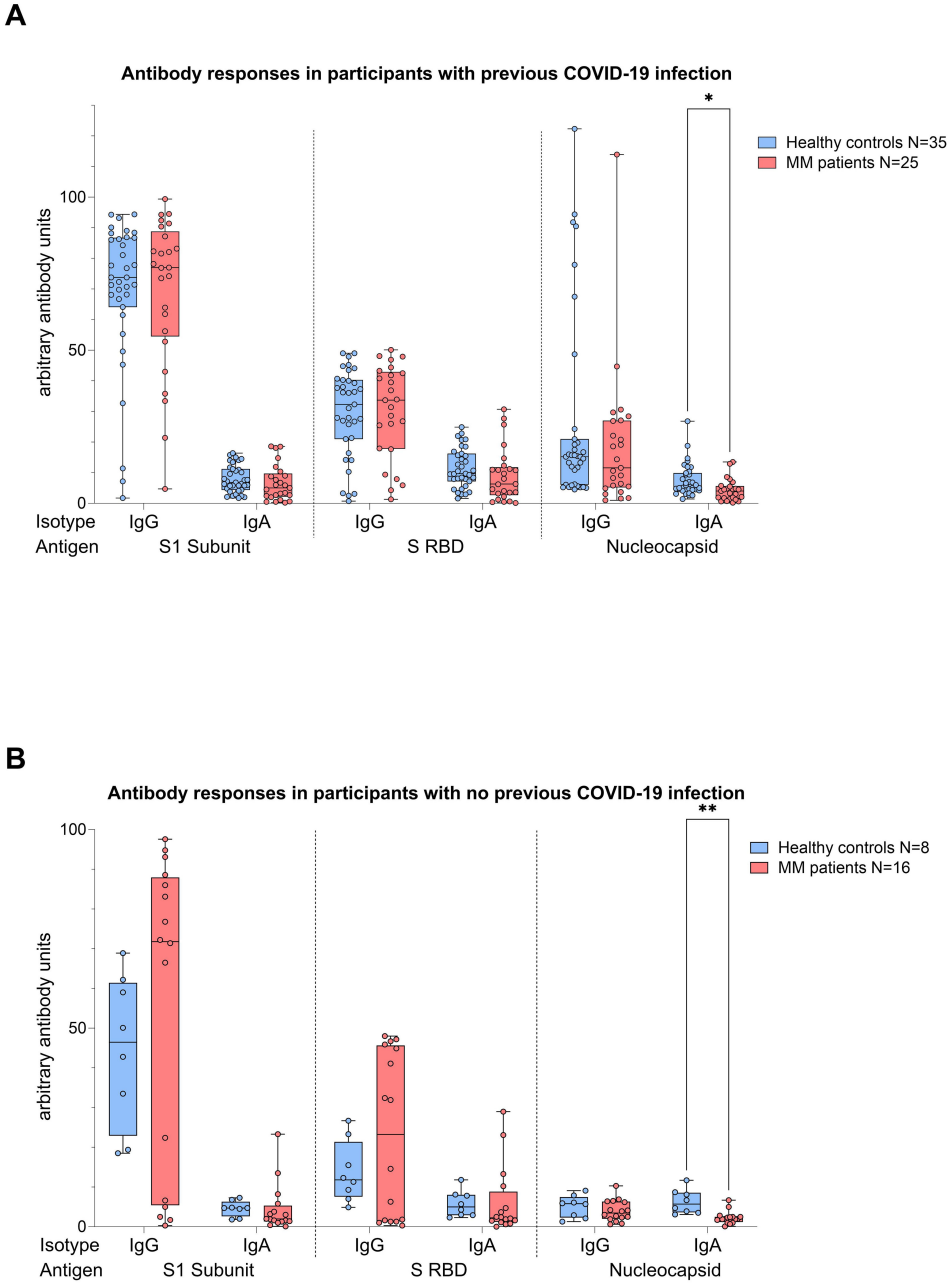
Boxplot comparing antibody responses [IgA, IgG] from healthy individuals (control group, in blue) to MM patients with ongoing or prior history of Lena intake (in red), dependent on their COVID-19 status. X-axis: antibody against SARS-CoV-2 spike protein (S1), spike-RBD and nucleocapsid protein (N). Y-axis: arbitary units of antibody levels. **(A)** (top): COVID-19 group, **(B)** (bottom): No COVID-19 group. The control group presented with significant higher N IgA levels (COVID-19 group: *p=0.022, No COVID-19 group: **p=0.001). Data is represented in a box plot diagram indicating media, Q1 and Q3 with min/max as whiskers.

Analyses performed independently of the COVID-19 status showed better antibody responses in healthy controls versus MM patients, also after balancing for vaccination (N IgA levels p<0.001, **Figure 4**). When analyses were performed taking COVID-19 status into account, higher N IgA responses in healthy controls remained robust (COVID: p=0.022, No COVID: p=0.001, **Figure 5**). The superior antibody responses of healthy controls in the originally defined ‘no Covid’ group were most likely attributable to asymptomatic viral exposure in this cohort. The observation in N IgA responses was by trend accompanied by enhanced levels of N IgG (p=0.078), Spike-1 IgA and Spike-RBD IgA (both p=0.069), all of them independent of the COVID-19 status. In contrast, Spike-1 IgG and Spike-RBD IgG were not significantly different but by trend showed higher antibody levels in MM versus healthy controls, excluding COVID-19 exposure history (mean S1 IgG: 44.2 vs. 40.88, mean S-RBD: 43.55 vs. 41.50, p=0.786). Regarding seroconversion rates in our cohort, we counted 85.4% and 93.0% seropositive MM patients and healthy individuals, respectively. The 14.6% (n=6) MM patients with seronegative profile presented the following accompanying T cell responses: 50% (n=3) did not elicit any S directed T cell response, and the other 50% (n=3) exhibited mixed T cell responses. All antibody results, measured against their defined thresholds, are listed in **Table 3**.

**Table 3:** Positive antibody responses measured against their defined thresholds.

Antibody target and isotype	MM group (in % above threshold)	Healthy group (in % above threshold)
Spike-1 IgG (= seroconversion)	85.4	93.0
Spike-1 IgA	19.5	27.9
Spike-RBD IgG	78.0	88.4
Spike-RBD IgA	14.6	25.6
Nucleocapsid IgG	26.8	44.2
Nucleocapsid IgA	0.0	4.7

Considering the significant age difference between the healthy and the MM group, we performed an age matching. Here, 11 controls and 11 MM patients got matched. Apart from the known significant difference in N IgA values (p<0.001), no new insights were gained regarding humoral and cellular responses. In addition, antibody and T cell responses did not differ based on the elapsed time since HDC/ASCT (in all comparisons p>>0.05). Similarly, we observed no difference in antibody and T cell responses based on the remission status of the patients (CR vs. no CR, in all comparisons p>>0.05).

### Correlation analysis in the whole cohort

Correlation analysis revealed a negative correlation between age and N act. CD8+ T cells (p=0.016, r = -0.373) in the MM group, while no association was observed among the healthy controls. Furthermore, no other significant correlations or trends between age and immune responses could be seen. Exploring the association between humoral and cellular responses, we identified different patterns comparing the healthy and MM group. Whereas antibody responses correlated significantly with N-specific T cell responses in healthy individuals, MM patients showed a significant correlation to S-specific T cell responses (**Supplementary Table S2**). Notably, also CD8^+^ cells and anti-spike RBD IgG showed a strong positive correlation (p=0.001, r=0.5) in MM patients.

### Vaccination responses in the myeloma group divided by Lena intake and Lena break

**Figure 6:**
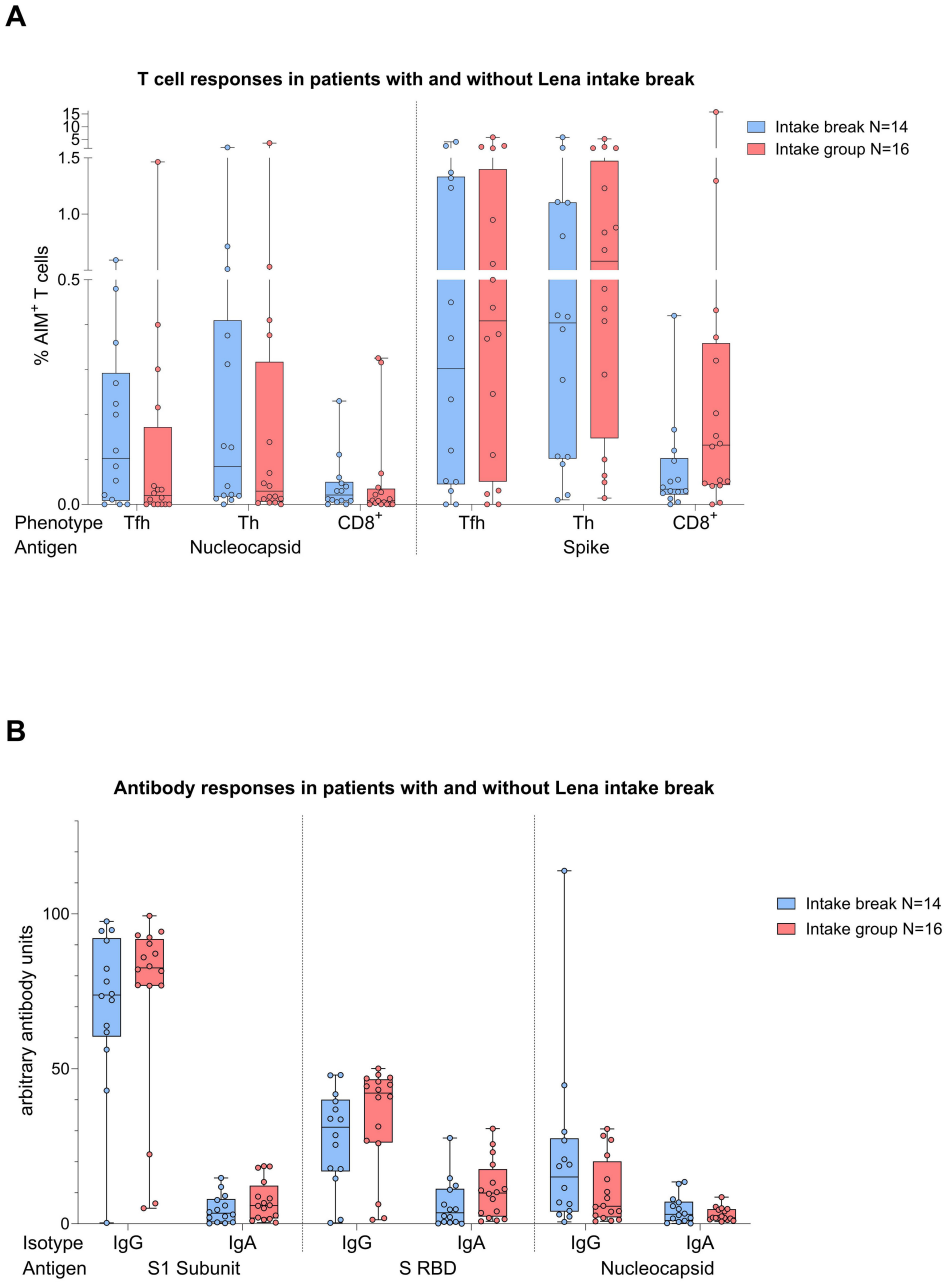
Boxplot comparing in **(A)** (top) T cell responses [CD4^+^ T follicular helper (T_FH_), CD4^+^ T helper cells (T_H_ cells), CD8^+^ T cells], and in **(B)** (bottom) antibody responses [IgA, IgG] from MM patients with Lena intake break (in blue) to MM patients with ongoing Lena intake (in red). X-axis: (top) T cell activation against SARS-CoV-2 nucleocapsid protein (N) and spike protein (S); (bottom) antibody against SARS-CoV-2 spike protein (S1), spike-RBD and nucleocapsid protein (N). Y-axis: (top) respective percentages (%) of activated T cells; (bottom) arbitary units of antibody levels. No significant differences could be found in group comparison analysis. Data is represented in a box plot diagram indicating media, Q1 and Q3 with min/max as whiskers.

Given the immunomodulatory effect of Lena on T cells and debates about intake breaks around vaccinations, we scrutinized the impact of Lena breaks (≥7 days) around the vaccination in our cohort. We had information about n=14 and n=16 MM patients who reported an intake break and continued intake during vaccination (intake group) in the questionnaire, respectively. From n=4 MM patients no information was provided. We found no significant differences regarding T cell (**Figure 6A**) and antibody (**Figure 6B**) responses between Lena intake and break group, *independent* of their COVID-19 status. T cell responses in the intake group, however, showed a trend towards higher S activated CD8^+^ T cell frequencies (T_FH_ p=0.802, T_H_ p=0.802, CD8^+^ p=0.235). Regarding N activated T cells, this trend was reversed. Detailed statistics are outlined in **Table 4**. Patients with continued intake had shorter times since last vaccination (p=0.024) by equal vaccination counts (p=0.099). In the intake group, more patients were less than two years under Lena maintenance, compared to the break group. No significant difference was detected in other potentially confounding factors such as age (p=0.47), gender (p>0.99) or time since last infection (p=0.24). Regardless of the break status, the time since the last infection was not different between groups (p=0.240). In correlation analysis we saw a negative correlation between spike RBD IgA and the duration of Lena break (p=0.016, r = - 0.435), as well as with S CD8+ (p=0.030, r = - 0.396) and a trend to significance with Spike-1 IgA (p=0.058) and spike RBD IgG (p=0.052). Indicating potentially reduced immune responses because auf Lena intake break.

**Table 4:** Comparison of T cell and antibody responses dependent on Lenalidomide intake or break around COVID-19 vaccination using Mann-Whitney U-Testing.

Response	Cell type	Mann-Whitney test: P-value
T cells	N T_FH_ N T_H_ N CD8 S T_FH_ S T_H_ S CD8	0.727 0.802 0.767 0.802 0.802 0.235
Antibodies	Spike-1 IgG Spike-1 IgA Spike-RBD IgG Spike-RBD IgA N IgG N IgA	0.667 0.666 0.573 0.528 0.666 0.689

## Discussion

In this work, we investigated the immune responses after SARS-CoV-2 vaccination or infection in MM patients with an ongoing or prior history of Lena maintenance therapy. Healthy individuals served as control group. Our main goals were (1) to investigate humoral and cellular vaccine responses, and (2) to identify the role of Lena in the context of vaccination in this specific population. The most important finding is that we observed comparable T cell responses to SARS-CoV-2 between MM patients and healthy controls in our experiments. In contrast, antibody responses were significantly weaker in the MM cohort. Alike previous studies, we underline the assumption, that humoral and cellular immunity are differently mediated in immunocompromised patients compared to healthy individuals and are not inevitably associated to each other (8, 15, 23–25). The following discussion of our results will provide important insights for a better understanding of this increasingly investigated topic. First, considering the quantitative superiority of vaccination counts in the MM group, MM patients with an ongoing or prior history of Lena intake even achieved better T cell responses in comparison to healthy individuals. Putting our results in context with previous studies on haematologic cancer patients, the T cell responses in the MM group are located in the upper part with 75.6%, compared to the wide range of 26% - 75.6% reported cellular responses (8, 9, 11, 26). In line with the better T cell responses of the MM group, we documented a higher rate of SARS-CoV-2 infections in the healthy group (80%) compared to the MM group (63.14%). As explanation, besides the better T cell vaccine response, one can also imagine a stricter behavioral protection of the immunocompromised group. But in addition, the significant older age in the MM group and the known associated immuno-senescence should have led to worse immune responses. To exclude any bias, we complemented a subgroup analysis with age-matched healthy individuals, which revealed unchanged good T cell responses in the MM group. The comparable T cell responses despite the higher age in the MM group suggest a potential for immunomodulatory therapy with Lena in MM patients around vaccination. However, it is to note that this subset was substantially smaller than the total cohort which reduces the robustness of these results.

The fact that there are scant and non-evidence-based recommendations regarding Lena intake around vaccinations prompted us to compare patients who paused the medication with those who did not. The impact of a Lena break ≥ 7 days around vaccination showed no negative or positive effect on T cell vaccine response in our cohort. The significant negative correlation between Spike RBD IgA and Spike CD8^+^ T cells with the duration of the Lena break (p=0.016 and p=0.030, respectively) argues against a positive effect of a break on antibody formation after vaccination. This could have been assumed, as Lena is known to have a negative effect on B cell or plasma cell proliferation in the context of MM therapy, potentially also on healthy plasma cells explaining reduced antibody responses. However, Van Oekelen et al. saw no significant influence of IMiD treatment on antibody vaccine responses (27). Our study results refute the notion of a negative effect of Lena intake on both humoral and cellular vaccine responses. Whether the IMiD could even have a positive effect on cellular responses remains to be further explored. Preclinical studies have convincingly demonstrated a positive influence of Lena on T cell functioning (28). This finding was also reproducible in clinical settings (29), seeing maximal vaccine efficacy when administered concomitantly with Lena (21, 28). Exemplarily, a study with pneumococcal vaccine documented higher antigen-specific T cell responses under Lena treatment (21). The reason for the lack of significant differences between the intake and break group in our study could be explained by low case numbers as well as the average Lena dose, which was lower than in the aforementioned study (9 mg/day in our cohort vs. 20 mg/day).

Furthermore, we want to draw attention to four minor discussion points. Taking a closer look to the factor age, a negative correlation between age and activated CD8^+^ T cells against N post-infection is worth mentioning. This observation could be explained solely by older age. But another suggestion is that infections may induce less protective immunity compared to vaccinations in the MM population. This consideration is supported by our correlation results, in which MM patients, unlike healthy individuals, showed only significant correlation between antibody responses to S act. T cell responses, whereas correlations to N act. T cells were absent. Herewith, our results reinforce the idea that vaccine-evoked cellular responses tend to be more robust than those triggered by infection. Existing data substantiate the added value of booster vaccinations in this vulnerable population to ensure adequate protection (18, 30, 31). Regarding the humoral responses in detail, an interesting observation was that Spike-1 IgG and Spike-RBD IgG levels were slightly higher in the MM group and therefore, stand in contrast to the collectively worse other antibody levels. Accordingly, IgG seroconversion rates were quite high in our MM group with 85.4%. In comparison, another trial could verify a seroconversion in only 57.8% of their patients with haematological malignancies (8). Here, it is important to remember the higher vaccination count in our MM cohort. Considering the overall picture, with higher N IgG and IgA, Spike-1 IgA and Spike-RBD IgA levels we stick to the theory of generally stronger humoral immune response in healthy individuals. Nevertheless, these results should be rechecked and validated in future trials. In the fourth and final point, we want to address the methodology of analyzing SARS-CoV-2 antibody responses. While our study measured antibody titers, we did not assess the neutralizing capacity of these antibodies, which is considered an accepted correlate of protection. However, multiple studies have demonstrated that serological responses strongly correlate with neutralizing titers (32–34), particularly when the time elapsed between vaccination and blood sampling is extended, as in our study. This was also shown in MM patients and precursor stages (31). Furthermore, serological antibody levels have been shown to correlate with reduced infection risk (35–37), a finding we also expect in our study cohort.

Strengths of the study are a comprehensive evaluation of T cell responses in healthy controls and MM patients. The used AIM assays are known to be successful in a range of previous studies in detecting antigen-specific T cells (38). Thus, we could accurately evaluate the power of MM patients’ immune systems. Caveats of our study include the sample size and the long recruitment period of 1.5 years. Additionally, the missing data on quantitative T cell responses and neutralizing capacity of the antibodies, as mentioned above, as well as no bone marrow evaluation lacks holism. For future research, we recommend increasing the sample size and, if possible, including bone marrow (BM) samples, which is considered as an immune niche of antigen-specific T cells. Therefore, T cell responses in the BM are said more robust and could be investigated in association to the surrounding microenvironment (21).

In sum, our findings are consistent with our expectations, underscoring the deficiency in antibody responses among MM patients following either exposure to the virus or vaccination. However, considering the satisfactory T cell responses despite prior or ongoing Lena therapy, our data support the notion that Lena does not influence this cellular-based response negatively. We anticipate that cellular responses are more robust when being vaccination-evoked then infection-triggered, pinpointing out the indispensability of vaccinations in MM patients for eliciting stronger and more reliable immune responses. Our observations are of immediate clinical relevance since currently no recommendations exist regarding the method of IMiD administration around vaccination, and several colleagues may – perhaps counterproductively – pause the drug to allow vaccine response. Acquired insight could be transferable to vaccination strategies for broader patient populations with immunosuppression. Lena and other IMiDs demonstrate growing importance in diverse cancer treatments. Therefore, we have a critical need for profound knowledge about their influence on the T cell compartment and more precisely, a need for understanding their influence on cellular-based vaccine responses. The working hypothesis for the future would be that Lena augments the global immune responsiveness and consequently, the efficacy of vaccines.

## Conclusion

Overall, these observations highlight significant differences in the immune capabilities of MM patients and healthy individuals. MM patients undergoing Lena maintenance therapy exhibit weakened antibody production but present equivalent T cell responses following SARS-CoV-2 vaccination in comparison to healthy individuals. This outcome showcases the importance of vaccination in MM patients and moreover, our results argue against a Lena break around the time of vaccination. Overall, T cell-based vaccine responses gain more importance in immunocompromised patients and should be further explored. Prospective clinical trials are essential to validate this potential and provide definitive evidence. Especially a double-blinded study comparing the vaccine responses in patients that take immunomodulatory drugs could further decipher potential benefits of those drugs in improving vaccine responses in vulnerable populations.

## Data Availability

The original contributions presented in the study are included in the article/**Supplementary Material**. Further inquiries can be directed to the corresponding author.
