# Supplementary material for: SARS-CoV-2 immune responses in patients with multiple myeloma and lenalidomide maintenance therapy
Source: Front Immunol. 2024 Dec 18;15:1510942. doi: 10.3389/fimmu.2024.1510942 (PMC11688394; doi:10.3389/fimmu.2024.1510942)
Supplement: Supplementary file 1 [file DataSheet1.pdf]

Supplementary Material

Supp. Material 1 translated questionnaire

Name ______________________

Date of birth ______________________

Do you have any known pre-existing diseases? ☐ yes ☐ no

➔ If yes, please specify: ______________________

Is your hematologic disease acute or recurrent? ☐ Acute ☐ Recurrent

Do you take any medication regularly? ☐ yes ☐ no

➔ If so, please specify? ______________________

➔ Do you take any immunomodulatory drugs (e.g., Lenalidomide)?

☐ yes ☐ no If yes, please specify: ______________________

Have you been vaccinated against the SARS-CoV-2 virus? ☐ yes ☐ no

➔ If yes, how often? ____________

➔ Please indicate the dates of each vaccination:

1st vaccination ____________ 2nd vaccination ____________

3rd vaccination ____________ 4th vaccination ____________

➔ Please specify which vaccines were used:

1st vaccination ____________ 2nd vaccination ____________

3rd vaccination ____________ 4th vaccination ____________

Were you taking any immunomodulatory drugs during the vaccination period?
☐ yes ☐ no

Have you had close contact to any COVID-19 positive individuals? ☐ yes ☐ no

Have you had a SARS-CoV-2 infection? ☐ yes ☐ no

➔ If yes, when? Please indicate the date of your first positive rapid test or PCR test.

Date of SARS-CoV-2 infection: ________________

➔ How long did your symptoms last? ________________

➔ How long after the acute infection did you feel completely recovered and free of symptoms?

Period „Long-COVID-symptoms" (if applicable) ________________

➔ On a scale of 1 to 10, please rate the severity of your disease. The table below can be used as a guide for classification. Empty fields in the symptom column can be used to add additional symptoms that may affect your subjective score.

Score: ________________

Score	Classification	Definition	Symptoms
**0**	**No SARS-CoV-2 Infection**	No SARS-CoV-2 infection detected	None
**1**	**Asymptomatic SARS-CoV-2 infection**	Positive SARS-CoV-2- PCR test or positive antigen- rapid test	None
**2**	**Mild disease**	No criteria indicating severe or critical infection	respiratory symptoms, cold-like symptoms, temporary changes in taste or smell, diarrhea
**3**			
**4**	**Moderate disease**	No criteria indicating severe or critical infection	Pneumonia (without O_2-supplementation), fever, severe headache, severe body aches, severe fatigue
**5**			
**6**			
**7**	**Severe disease**	Severe pneumonia	Defined as: SpO2 < 90-94%, respiratory frequency > 30/min, radiological evidence for pneumonia
**8**			
**9**	**Critical disease**	ARDS, sepsis, septic Shock +/- Multi organ failure	Mechanical ventilation required or other organ support or administration of vasopressors
**10**			

Modified classification based on the clinical classification of SARS-CoV-2- infections of the Robert Koch Institute

Supp. Material 2

Supplemental Table 1: *Anti-human antibodies used for activation induced marker (AIM) assay.*

Fluorochrome	Antigen	Clone	Dilution	Product number
VioBright FITC	CD137	REA765	1:50	130-110-765
APC-Vio770	CD69	REA824	1:200	130-112-805
VioGreen	CD14	REA599	1:800	130-110-583
VioGreen	CD19	REA675	1:400	130-114-175
PerCP-Vio700	CD8	REA734	1:400	130-110-820
VioBright R720	CD4	REA623	1:400	130-127-398
(CXCR5)-APC	CD185	REA103	1:200	130-122-926
PE	CD25	REA945	1:200	130-115-534
VioBlue	CD3	REA613	1:200	130-114-519
(OX40)-PE-Vio770	CD134	REA621	1:100	130-120-723
Viobility 405/520 Fixable Dye			1:800	130-109-814

Supp. Material 3

Supplemental Table 2: *Correlation of T cells and antibodies in MM and Healthy group.*

**MM group**		**Healthy group**	
**S T_{FH}**	Spike-RBD IgG, p=0.042, r= 0.320	**N T_{FH}**	Spike-1 IgG p=0.001, r= 0.480<br>Spike-1 IgA p<0.001, r= 0.499<br>Spike-RBD IgG p=0.003, r= 0.438<br>Spike-RBD-IgA p<0.001, r= 0.499<br>Nucleocapsid IgG p=0.049, r= 0.302
**S T_H**	Spike-RBD IgG, p=0.013, r= 0.383<br>Spike-RBD IgA, p=0.043, r= 0.317	**N T_H**	Spike-1 IgG p=0.011, r= 0.383<br>Spike-1 IgA p<0.001, r= 0.496<br>Spike-RBD IgG p=0.026, r= 0.340<br>Spike-RBD IgA p<0.001, r= 0.490<br>Nucleocapsid IgA p=0.002, r= 0.450
**S $CD8^+$**	Spike-RBD IgG, p=0.001, r= 0.500<br>Spike-RBD IgA, p=0.048, r= 0.311	**N $CD8^+$**	Spike-1 IgA p=0.011, r= 0.386<br>Spike-RBD IgA p=0.035, r= 0.323

Supp. Material 4

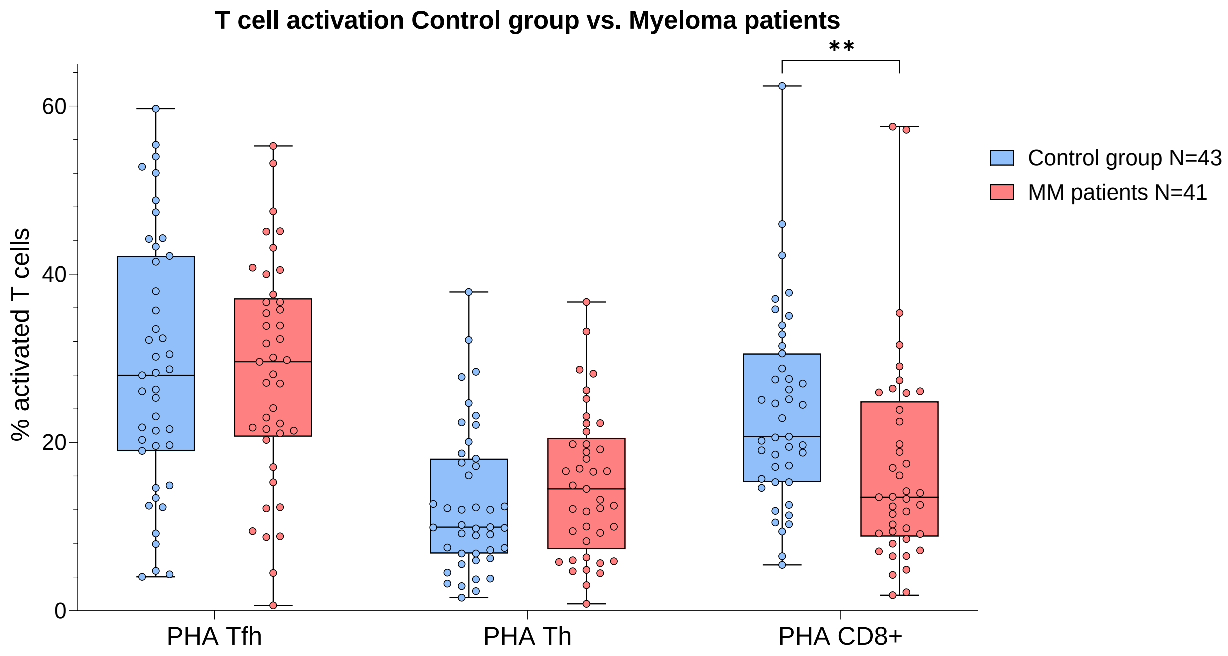

Supplemental Figure 1*: Boxplot comparing T cell responses [CD4^{+} T follicular helper (T_{FH}), CD4^{+} T helper cells (T_{H} cells), CD8^{+} T cells] from healthy individuals (control group, in blue) to MM patients with ongoing or prior history of Lena intake (in red). X-axis: T cell activation against PHA. Y-axis: respective percentages (%) of activated T cells. The control group presented with significant higher PHA CD8^{+} T cell levels (** p=0.005).*
